# Taxonomy, carcinogenic mechanisms, and advanced molecular diagnosis of Fusobacterium nucleatum in colorectal cancer: from bench to clinical practice

**DOI:** 10.3389/fimmu.2026.1840240

**Published:** 2026-06-29

**Authors:** Bin Li, Yifei Dong, Yifei Dai, Mengpei Zhang, Zhenwei Zou, Hai Yang

**Affiliations:** 1 Joint Research Center for Nanomedicine in Digestive and Metabolic Diseases, The Eighth Hospital of Wuhan (Wuhan Anorectal Hospital), Wuhan, Hubei, China; 2 Pathology Center, Sinopharm Medical Laboratory (Wuhan) Co., Ltd., Wuhan, Hubei, China; 3 National Engineering Research Center for Nanomedicine, College of Life Science and Technology, Huazhong University of Science and Technology, Wuhan, Hubei, China; 4 Cancer Centre of Union Hospital, Tongji Medical College, Huazhong University of Science and Technology, Wuhan, Hubei, China

**Keywords:** carcinogenesis mechanism, colorectal cancer, *Fusobacterium nucleatum*, noninvasive molecular diagnosis, taxonomic reclassification and distribution

## Abstract

Fusobacterium nucleatum (Fn) is recognized as a novel oncogenic organism closely correlated with colorectal cancer (CRC). Fn can induce gut dysbiosis and promote tumorigenesis through various mechanisms, including activation of oncogenic signaling, modulation of the immune microenvironment, metabolic reprogramming, and epigenetic regulation. Although recent technological breakthroughs have yielded high-resolution Fn detection indicators, clinical translation remains compromised by subspecies heterogeneity, emerging taxonomic updates, and non-standardization of diagnostic platforms. This review integrates recent developments regarding Fn pathogenic mechanisms and taxonomic identification in CRC, including genetic heterogeneity analysis at the species and subspecies levels, elucidation of multi-dimensional oncogenic molecular mechanisms, and application of high-resolution detection technologies. Special focus is placed on advancing these findings toward early noninvasive diagnostic markers. Additionally, we discuss the limitations of current studies, technical bottlenecks, and potential directions for future research to pave the way for standardized clinical screening.^C^

## 1 Introduction

Fusobacterium nucleatum (Fn), a Gram-negative anaerobe indigenous to the oral cavity, has been implicated as a potential oncobacterium that preferentially colonizes colorectal cancer (CRC) tissue and may actively contribute to tumorigenesis, though its causal role remains under investigation (1, 2). Over the past decade, accumulating molecular epidemiological evidence has revealed that Fn enrichment correlates with dysregulation of oncogenic signaling pathways, alteration of the tumor immune microenvironment, activation of metabolic reprogramming, and participation in genetic alterations, indicating that its presence is not merely associative but functionally contributive.

Mechanistically, Fn orchestrates multi-layered oncogenic programs: It activates NF-*κ*B and Wnt/*β*-catenin axes to accelerate cell proliferation (3, 4), remodels the immune microenvironment toward an immunosuppressive phenotype that attenuates immune checkpoint blockade efficacy (5), increases tumor progression (6–8), and reprograms cellular metabolism to favor fatty-acid oxidation and glycolysis, thereby enhancing cancer stem cell self-renewal (9). Concurrently, Fn influences the regulation of tumorigenesis by inducing changes in DNA methylation, thus contributing to tumorigenesis and development (10, 11). However, conflicting epidemiological data indicates Fn is present in only a subset of CRC patients (12), and confounding factors such as oral hygiene status and concurrent microbial infections may influence this association (1). Additional large-scale longitudinal studies and mechanistic investigations are needed to establish a definitive causal relationship.

To investigate the role of Fn in these mechanisms, technological advances have transitioned Fn detection from culture-based assays and 16S rRNA profiling to high-resolution *in-situ* hybridization, quantitative PCR and next-generation sequencing (NGS), enabling species- and subspecies-level quantification with single-copy sensitivity (13, 14). These innovations have not only refined our understanding of Fn-host interactions but have also laid the technical groundwork for exploiting Fn as a noninvasive biomarker and a therapeutic target in CRC.

This review discusses recent advances in Fn taxonomy, molecular detection, and therapeutic targeting, and how these can assist in addressing three major challenges: (1) delineating the mechanistic contributions of Fn subspecies to CRC pathogenesis; (2) optimizing noninvasive Fn detection for clinical screening; and (3) developing precision therapeutic strategies based on Fn status. Through the comprehensive pathway linking basic research to clinic, we intend to provide insights for novel CRC precision medicine therapeutic approaches.

## 2 Biological characteristics and distribution patterns of Fn

### 2.1 Fn classification system and genome characteristics

Historically, Fusobacterium nucleatum (Fn) sensu lato was considered a single species comprising four primary subspecies: nucleatum (Fnn), polymorphum (Fnp), vincentii (Fnv), and animalis (Fna), with fusiforme recently synonymized into Fnv (15–17). This taxonomy has long been a subject of debate due to the complex phenotypic and genotypic diversity across the subspecies complex (18). Recent genomic advancements have revolutionized this classification. While early phenotypic traits like bile sensitivity and acid production provided initial differentiation (**Table 1**), modern molecular tools such as 16S rRNA sequencing and multi-locus sequence analysis (MLSA) of housekeeping genes (e.g., rpoB) have exposed the limitations of traditional nomenclature (19). Specifically, genome-wide average nucleotide identity (ANI) and core genome phylogenetic analysis have emerged as the ‘gold standard’ for resolution. Building on the proposal by Kook et al. (17) to elevate subspecies to the species level-a move now endorsed by the NCBI database—subsequent studies have clarified the distinct identities of these lineages (20). A critical development involves the Fna clade. While Zepeda-Rivera et al. (19) initially identified two distinct clades, Fna C1 and Fna C2, further research has confirmed they represent separate species: Fna C1 has been reclassified as F. watanabei (21), whereas C2 corresponds to F. animalis (22). Notably, Fna C2 (now F. animalis) is the primary driver of adverse clinical signals in colorectal cancer, correlating with metastasis and early relapse, whereas Fna C1 (F. watanabei) remains largely associated with oral health (23). Most recently, a comprehensive study integrating ANI and phylogenetics has resolved long-standing taxonomic ambiguities, confirming that F. vincentii, F. polymorphum, F. nucleatum sensu stricto, and the two Fna clades are indeed distinct, independent species (24).

**Table 1:** Fn classification and phenotypic characteristics.

Subspecies name	Phenotypic characteristics
*F. nucleatum* subsp. *nucleatum*	Produces butyric acid, sensitive to bile
*F. nucleatum* subsp. *polymorphum*	Produces acetic acid, which is highly resistant to bile
*F. nucleatum* subsp. *vincentii*	Cell morphology is spindle-shaped (*fusiform*)
*F. nucleatum* subsp. *animalis*	Highly resistant to bile
*F. nucleatum* subsp. *fusiforme* (now included in *vincentii*)	Cell shape elongated

Overall, Fn subspecies differentiation and its genomic characteristics play a key role in the pathogenesis of CRC. Understanding these characteristics will provide new ideas and strategies for early diagnosis and targeted treatment of CRC.

### 2.2 Distribution patterns of human body and ecological niche characteristics

**Figure 1:**
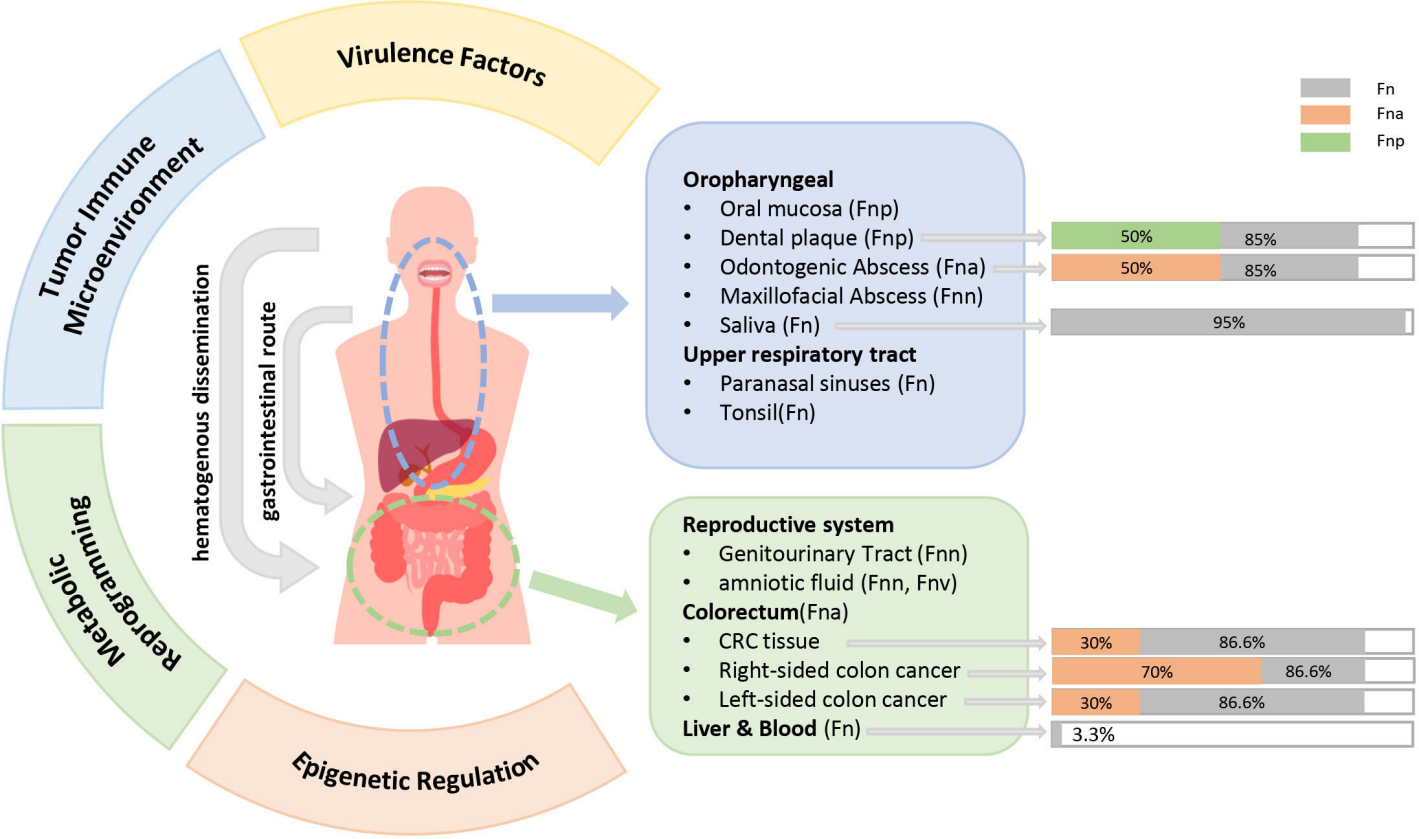
Distribution of *Fusobacterium nucleatum* subspecies in humans, inter-site transmission routes, and pathogenicity-associated factors.

With the taxonomic framework now more granular, attention turns to where and how these subspecies establish residence within the human host. Fn occupies a unique ecological niche in the human microbiome (**Figure 1**). In the oral environment, Fn is an important component of normal flora, and the detection rate in dental plaque exceeds 80%, of which the Fnp subspecies is the most common (accounting for about 50%). Disease disrupts this balance: when odontogenic abscesses form, bile-resistant Fna supplants Fnp and dominates the lesion (accounting for 50%) (25), illustrating that subspecies abundance is context-dependent rather than static. Saliva, which is continuously bathed in plaque-derived microbes, yields Fn in 95% of individuals (23), and strain-level analysis reveals that 75% of colorectal cancer patients carry identical genotypes in both saliva and tumor tissue−an early clue to the oral−CRC axis (26). Beyond the mouth, Fn traverses anatomical boundaries, upper respiratory sites such as tonsils and sinuses harbor the organism; sinus Fn may be one of the causes of brain abscess, although pus and biopsy specimens show comparable loads, suggesting passive carriage rather than active proliferation (27, 28). Genitourinary niches present a different picture: Fnn and Fnv are repeatedly isolated from amniotic fluid of preterm deliveries with amniotic fluid culture in premature women with intact fetal membranes (29, 30). In addition to facial abscesses, Fn strains in these locations are most likely to originate from the oral cavity because they are highly similar or consistent with the patient’s oral strains (13, 31, 32).

The gastrointestinal tract, however, represents the most clinically significant secondary habitat. In stool and colorectal cancer (CRC) samples, the detection rate of Fn in CRC patients was significantly increased. A meta-analysis including 627 CRC patients and 619 healthy controls showed that the fecal Fn abundance in CRC patients was significantly increased (19). In addition, Fn is also significantly increased in CRC tumor tissue, with a detection rate of 30%-86.6% (significantly higher than <10% in normal colon tissue). The proportion of Fn forming a biofilm in right colon cancer reaches 91.7% (58.7% in left colon cancer) (33). Multicenter cohort studies further confirmed that 70% of Fn-positive CRCs are located in the right colon and only 30% are located in the left colon (34). This phenomenon may be due to the fact that the right colon is responsible for the absorption of water and electrolytes, where the luminal content remains liquid and has a high pH, which is more conducive to the colonization and survival of oral anaerobic bacteria (such as Fn). The mucus layer is thin, making it easy for bacteria to contact and adhere to epithelial cells; moreover, the high concentration of bile acids is consistent with the enrichment of bile acid-resistant Fna subspecies in CRC.

Dissemination to remote organs is rare but informative for Fn. Fn was not detected in normal liver tissues (35), but 42% (92/218) of colorectal cancer (CRC) liver metastasis specimens harbored Fn DNA (7), suggesting that Fn may translocate to the liver along with CRC cells and persist in the metastatic niche. Fn can also be detected in blood, but the detection rate is extremely low: the positive rates of simultaneous detection of Fn DNA in saliva, tumor tissue and blood were 93.3%, 76.7% and 3.3%, respectively (only 1 out of 30 cases was positive) (36), underscoring tissue tropism rather than systemic circulation.

In summary, Fn primarily colonizes accessible anatomical sites (oral cavity, upper respiratory tract, genitourinary tract, right colon) and is detected in internal tissues, where it is predominantly associated with disease metastasis or infiltration (e.g., liver, blood system). However, classification of Fn at most tissue sites remains unclear, hindering the differentiation of diseases based on Fn subspecies.

### 2.3 Transfer pathways and colonization mechanisms from oral cavity to colorectum

Having mapped the preferential niches of Fn across human body sites, the next logical question is how the organism travels from its oral stronghold to the colorectal mucosa. Convincing evidence now indicates that >40% of CRC patients harbor identical Fn strains in both saliva and tumor tissue (26), thereby pointing to the oral cavity as the primary source. To bridge these distant anatomical regions, two non-mutually exclusive pathways have been proposed.

The first, and currently favored, route is hematogenous dissemination. Transient bacteremia triggered by routine chewing, dental procedures or everyday oral hygiene, which provides repeated opportunities for Fn to enter the systemic circulation (37). Once in the bloodstream, the bacterium employs its surface adhesion Fap2 to engage Gal-GalNAc carbohydrates that are overexpressed on colorectal vascular endothelium, facilitating both adhesion and transendothelial migration (38). Concurrently, Fn activates the TLR4/NF-*κ*B axis in endothelial cells, thereby increasing vascular permeability and further promoting its own extravasation toward the colorectal wall (39).

Complementing this vascular highway, an enteric route has also been validated. In experimental models, daily gavage of Fn into *Apc^min/^*^+^ mice yielded larger and more numerous colonic tumors compared with controls, and qPCR and FISH confirmed the presence of the introduced strain within tumor tissues (40). Although this pathway proves that simple swallowing can deliver viable bacteria to the large intestine, its efficiency is inherently lower than that of the hematogenous route. Mechanistically, reduced gastric acidity-whether pharmacologically induced by proton-pump inhibitors or pathologically present in inflammatory bowel disease-together with a compromised intestinal barrier allows orally derived Fn to survive gastric passage and establish residence in the colorectum (41).

Taken together, these findings outline a dual-portal model in which hematogenous spread provides the dominant conduit for rapid, targeted colonization, while the gastrointestinal route offers a supplementary, albeit slower, entry path. Understanding the relative contribution of each mechanism will be critical for designing interception strategies aimed at breaking the oral-CRC axis. Based on these insights into Fn dissemination and colonization, the following sections will provide an in-depth exploration of the impact of Fn on the development and progression of intestinal cancer and how specific virulence factors and subspecies characteristics translate into oncogenic phenotypes.

## 3 Progress in the molecular mechanism of Fn promoting the occurrence and development of CRC

### 3.1 Activation of oncogenic signaling pathways mediated by virulence factors

**Figure 2:**
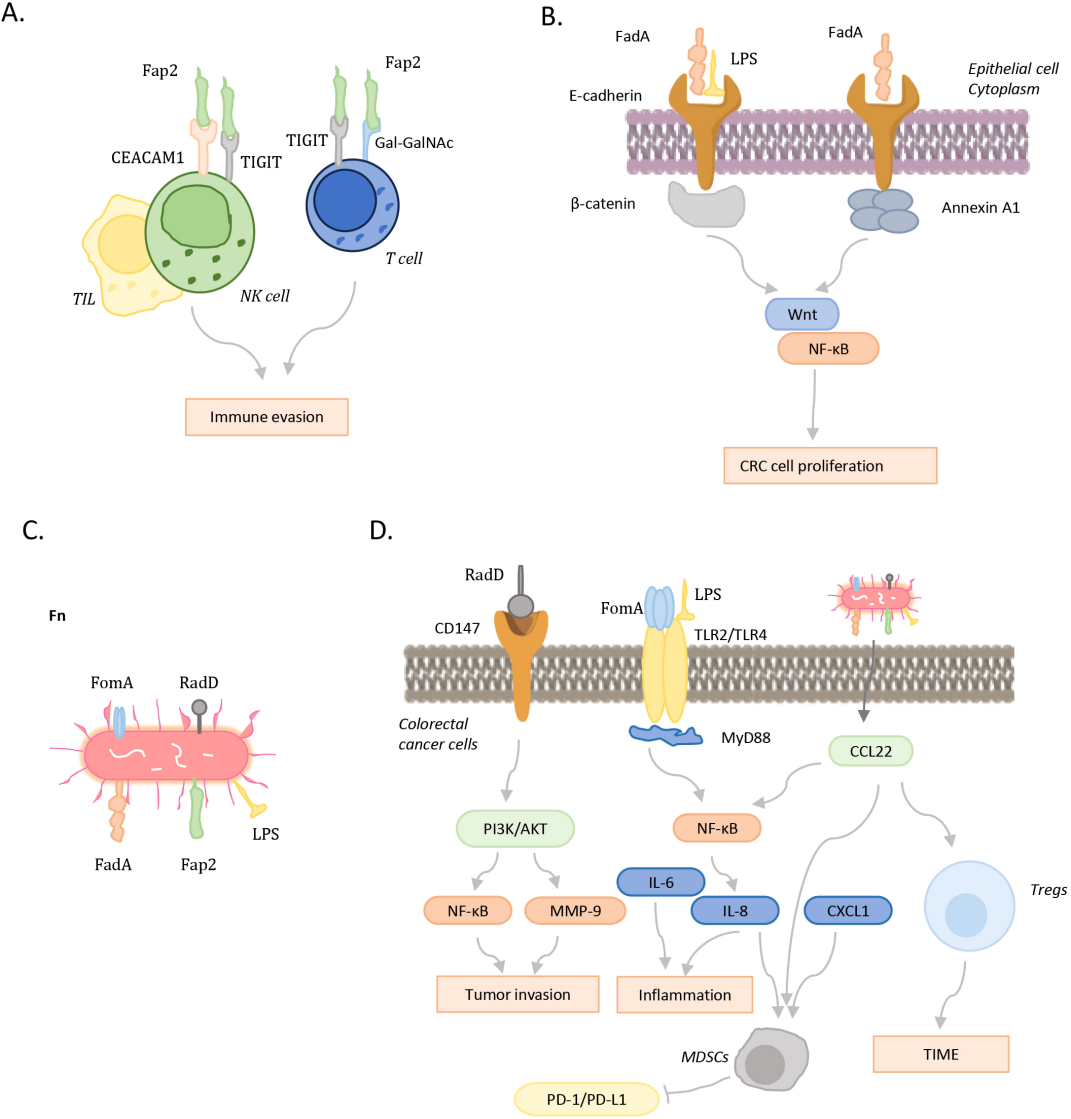
Signaling pathways and tumor immune microenvironment by mediated virulence factors. **(A)** Potential interactions of Fap2 with immune cells. Fap2 mediates immune evasion by binding to various receptors on T cells, NK cells, and tumor-infiltrating lymphocytes (TILs). **(B)** FadA interacts with the Ecadherin receptor on epithelial cells, activating *β*-catenin and Annexin A1, which subsequently triggers the Wnt/NF-*κ*B signaling pathway to promote tumor cell proliferation. **(C)** Fn schematic diagram. **(D)** RadD and FomA promote tumor invasion and inflammation by binding to Colorectal cancer cells receptors and activating PI3K/AKT and NF-*κ*B signaling pathways, recruiting MDSCs to disrupt PD-1/PD-L1 signaling axis. CCL22 expression is significantly upregulated in Fn-infected CRC cells, demonstrates a potential regulatory relationship with the NF-*κ*B signaling pathway. CCL22 interacts with Tregs, driving Tumor Immune Microenvironment (TIME).

Fn is widely distributed in colon cancer tissues and contributes to the development of colorectal cancer. Like other pathogens, Fn can express various surface adhesion proteins, which allows it to attach itself effectively to tumor cells (**Figure 2C**).

FadA, a key virulence factor, binds E-cadherin or Annexin A1 to activate the *β*-catenin signaling pathway, promoting CRC cell proliferation, tumor growth, and progression (42–44) (**Figure 2B**). FadA also inhibits apoptosis pathways and thereby increase the resistance of tumor 186 cells to chemotherapy.^D^

Another important pathogenicity factor is the Fap2 protein, which primarily suppresses the host’s immune response and promotes immune escape from tumors. Fap2 exerts its effect by interacting with Gal-GalNAc glycans on the surface of T cells. This binding leads to two main outcomes: it promotes the concentration of Fn in the tumor microenvironment (TME) while inhibiting the activity of T cells, which reduces the immune system’s ability to attack tumor cells and leads to an immune escape mechanism (45, 46). In addition, the immunosuppressive effect of Fap2 also extends to NK cells and tumor-infiltrating lymphocytes (TILs), which further suppress the host’s antitumor immune response (8). In patients with CRC the extent of Fap2 expression is closely associated with poorer clinical outcomes (47) (**Figure 2A**).

Fn is an adhesive bacterium that expresses several adhesion factors that mediate co-aggregation and biofilm formation, such as FomA and RadD. FomA, a ligand of TLR2/TLR4, activates the MyD88/NF-*κ*B pathway to induce IL-8 secretion, creating a chronic inflammatory microenvironment (40, 48); RadD interacts with the CD147 receptor and initiates the PI3K/AKT pathway, amplifying NF-*κ*B and MMP-9 expression, increasing tumor invasion (46) (**Figure 2D**). In addition, LPS binds to TLR4, and FadA activates NF-*κ*B and *β*-catenin signaling pathways, promoting inflammatory responses and colorectal cancer cell proliferation (1, 5) (**Figure 2D**).

Although these virulence factor-mediated signaling pathways establish the oncogenic potential of Fn, the impact of Fn is not limited to direct regulation of tumor cells but can also comprehensively reshape the tumor immune microenvironment.

### 3.2 Remodeling of the tumor immune microenvironment

Fn mediates immune evasion through direct inhibitory interactions and microenvironmental remodeling: it impairs NK cell cytotoxicity via Fap2-TIGIT binding (31) (**Figure 2A**) and suppresses T-cell function by downregulating adaptive immunity via the Wnt/*β*-catenin pathway (49). Additionally, Fn induces apoptosis of T cells, inhibits their proliferation, and reconstructs TME to create an immunosuppressive niche.

Beyond direct immunosuppression, Fn recruits CD11b-derived myeloid suppressor cells (MDSCs), which are often correlated with a poor prognosis and drug resistance (31). In *Apc^min/^*^+^ mice, Fn suppresses antitumor immunity and colorectal cancer development (50). Fn enhances an immunosuppressive TME by actively attracting MDSCs and tumor-associated macrophages. This organism employs numerous strategies to avoid detection by the immune system: 1). Suppression of NK cells: Fn activates TIGIT to inhibit NK cells, preventing them from killing CRC cells. 2). Fn activates CEACAM1 on immune cells and inhibits T-cell and NK-cell functions. 3). Disruption of iNKT cell activity leads to Fn-induced immune evasion, as iNKT cells have anti-tumor activity; 4). Fn enhances the production of chemokines such as IL-8 and CXCL1 which recruit MDSC. Therefore, PD-1/PD-L1 signaling may be dysregulated (51) (**Figure 2D**). The immunosuppressive milieu is further reinforced by regulatory T cell (Treg) infiltration. Clinical studies demonstrate a positive correlation between intratumoral Fn abundance and Treg prevalence, implicating this pathogen in the construction of a tolerance-permissive TME that undermines antitumor immunity (7, 52) (**Figure 2D**).

Fn interacts with host cells by binding to cell wall components at the toll-like receptor 4 (TLR4), thereby activating the MyD88 signaling pathway. This interaction leads to the release of pro-inflammatory cytokines, including IL-6 and IL-8 (53, 54), (**Figure 2D**) IL-8 further amplifies this inflammatory milieu by recruiting tumor-associated macrophages, thereby establishing a self-perpetuating cycle that supports malignant progression (55).

Overall, Fn reconstructs the tumor immune milieu through tripartite mechanisms: suppression of cytotoxic T/NK cells, recruitment of immunosuppressive MDSCs and TAMs, and sustained inflammatory cytokine production. Combination therapies that target these signaling pathways could represent a promising strategy for the later treatment of colorectal cancer. Beyond immunomodulation, Fn profoundly alters the metabolic landscape of the TME^D^.

### 3.3 Metabolic reprogramming and microbial crosstalk

**Figure 3:**
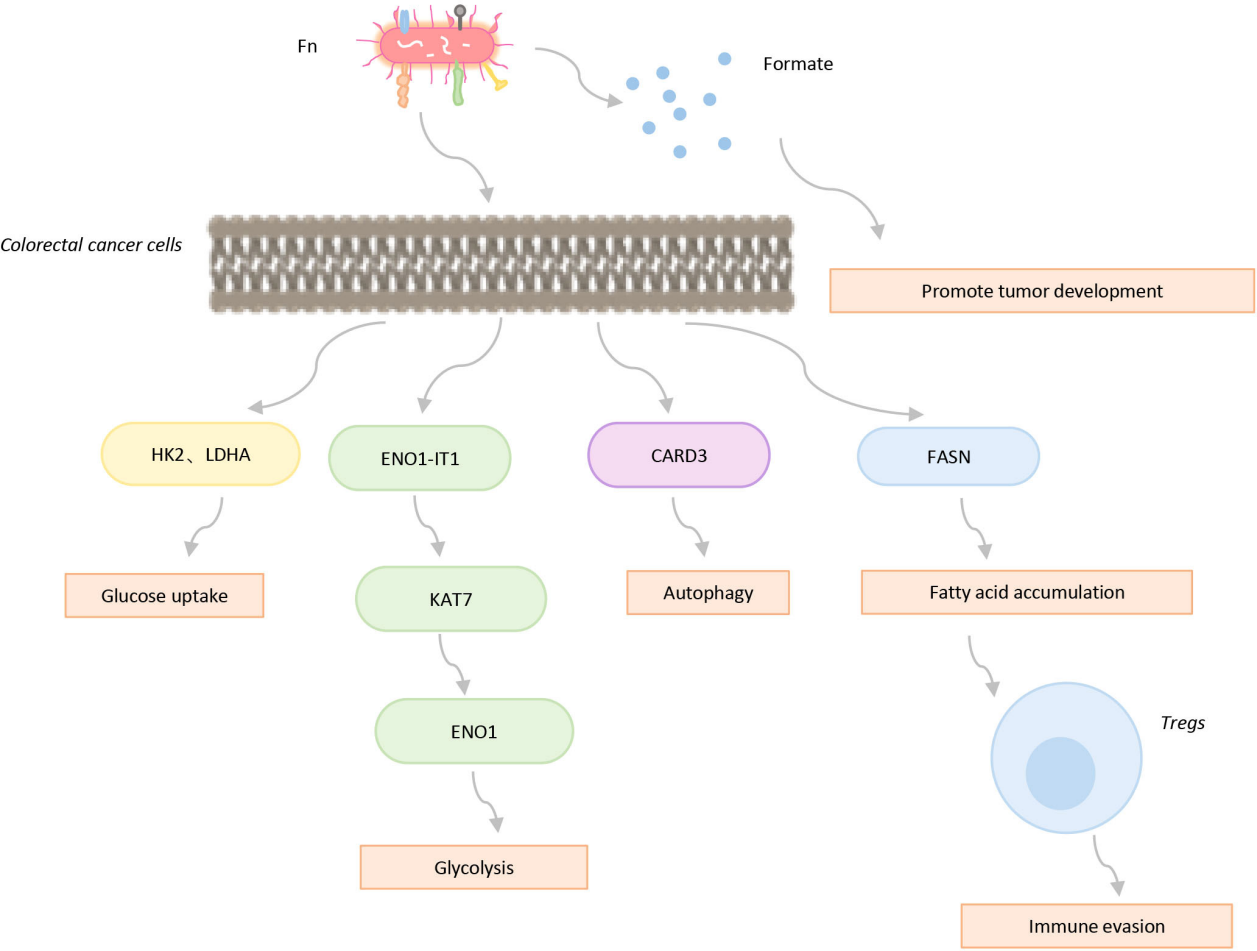
Metabolic reprogramming induced by Fusobacterium nucleatum in colorectal cancer. The metabolic byproduct formate released by Fn exacerbates CRC progression. In Fn-infected CRC cells, the expression of HK2 and LDHA is upregulated, accompanied by increased glucose uptake. Fn upregulates ENO1-IT1, activating the KAT7 acetyltransferase to promote ENO1 expression and enhance glycolysis. Fn also induces autophagy through upregulation of CARD3, supporting cancer cell survival and metastasis via nutrient recycling. Additionally, Fn upregulates FASN, facilitating fatty acid accumulation to provide a survival advantage for Tregs, thereby promoting immune evasion.

Fn plays an important role in the etiology of colorectal cancer and mediates metabolic reprogramming, which alters the energy metabolism of tumor cells. Ternes et al. showed that formate exacerbates tumor progression and directly promotes tumorigenesis (56) (**Figure 3**). Liu et al. further characterized the metabolic profile of CRC-associated bacteria (including Fn) and elucidated how their metabolites contribute to CRC development (57). Collectively, these results demonstrate that Fn metabolites, especially formate, have a regulatory impact on TME remodeling. Recent insights brought to light by Zhou et al. revealed that arginine metabolism enhances Fn stress resistance, facilitating bacterial colonization and tumor modulation (58). This metabolic change may help bacteria to infect and modulate tumor tissues. Leveraging synthetic microbial communities, the same group further demonstrated ecological control over Fn infection in CRC models (59). These results highlight the potential of a therapy that targets microbial metabolism to regulate the role of Fn.

At the cellular level, Fn infection upregulates glycolytic enzymes HK2 and LDHA to enhance glucose uptake (36),while inducing ENO1 expression to drive glycolytic flux (9). Furthermore, Fn promotes the expression of ENO1-IT1, which recruits KAT7 acetyltransferase, promoting ENO1 transcription. Fn-driven modulation of autophagy contributes to metastasis, chemoresistance, and epithelial-mesenchymal transition (EMT), enhancing cancer cell migration and metastasis by facilitating nutrient recycling and stress adaptation in the TME (60). For instance, Fn infection activates autophagy signaling by upregulating CARD3 expression, a key mediator of autophagosome formation. Additionally, Fn increases the accumulation of fatty acids, which provides a survival advantage for fatty acid oxidation-dependent Tregs and stabilizes FOXP3 expression via Warburg-associated lactate production (61) (**Figure 3**).

Fn not only has inherent carcinogenic ability but also acts synergistically with commensal bacteria like Escherichia coli to disrupt the intestinal mucosal barrier. This synergistic interaction exacerbates epithelial damage and barrier dysfunction, facilitating carcinogen access to the intestinal mucosa and consequent genomic instability (62, 63).In addition, Fn metabolites may leverage metabolites from other microbiota in the gut to change gut microenvironments to benefit tumors. Fn may reduce beneficial gut bacteria, thereby disturb microbial homeostasis and promote a favorable TME (64). Beyond these metabolic and ecological interactions, emerging evidence indicates that Fn can induce heritable epigenetic modifications that permanently alter host cell phenotypes.

### 3.4 Epigenetic regulation and metastasis promotion

Epigenetic regulation plays a crucial role in the etiology of colorectal cancer, especially during tumor metastasis. Fn infection has been shown to cause hypermethylation of the promoter region of tumor suppressor genes (such as CDKN2A), which affects gene expression and cancer progression (65). Hypermethylation generally inhibits the transcriptional activity of the major tumor suppressor genes, thus promoting the growth and metastasis of tumor cells. Understanding this mechanism is of great importance for understanding the development and development of colorectal cancer (66).

In addition, Fn infection was found to increase miR-21 expression and promote the transition between epithelium and mesenchyma (EMT) and distant metastases of cancer cells. EMT is a crucial step in which cancer cells acquire invasive and metastatic capabilities, and the rise in miR-21 is closely linked to a poor prognosis in various cancers. miR-21 increases cancer cell migration and invasion by regulating the expression of multiple downstream target genes, ultimately leading to tumor metastases and recurrence (67). In colorectal cancer, Fn infection not only promotes the proliferation and movement of tumor cells, but also alters the biological properties of the tumor by regulating miR-218-5p and its target genes (such as BIRC5 and DDX21), thereby exacerbating the severity of the disease (68, 69).

In summary, Fn significantly influences the metastasis process of colorectal cancer by inducing methylation of tumor suppressor genes and increasing miRNA expression. These results may shed light on the early detection of tumors and targeted therapies. Future studies should investigate the possibility of improving the prognosis and treatment response of colorectal cancer patients through the regulation of these epigenetic changes. These insights into the mechanisms of Fn-driven carcinogenesis lay the foundation for developing targeted diagnostic and therapeutic strategies. It is crucial to translate genotyping technology and molecular detection methods into clinical applications and apply them to colon cancer diagnosis, prognosis and personalized treatment.

## 4 Fn genotyping: clinical diagnosis and treatment guidance

### 4.1 Genotyping detection technology and subspecies-specific identification

In view of the multi-layered Fn−CRC interactome that spans signaling, immune and metabolic axes, there is an urgent need for high-resolution, rapid, cost-effective and noninvasive translational platforms to seamlessly bridge mechanistic dissection with clinical validation through genotyping.

In the research of CRC, the genotyping detection technology of Fn has undergone a progressive development from broad-spectrum screening to targeted identification. Metagenomic sequencing, particularly NGS, has enabled global discovery of Fn in the tumor microenvironment was achieved through metagenomic sequencing. Its unbiased characteristic can simultaneously identify bacterial species, subspecies and symbiotic microbial communities, providing key evidence for revealing the co-evolutionary mechanisms of Fn and CRC (32, 70). However, the high cost and technical complexity of NGS have limited its clinical translation, prompting researchers to turn to more targeted molecular typing strategies. Against this background, 16S rRNA gene sequencing for the detection of Fn subspecies emerged. However, due to the small differences in 16S rRNA gene sequences among various Fn subspecies (few SNPs), the resolution and confidence are relatively low when used to distinguish subspecies (13). Targeted amplicon sequencing (TAS) achieves in-depth analysis at the subspecies level by focusing on highly variable genes Notably, FrpoB-seq (a targeted amplicon sequencing technique based on rpoB) has been recently developed for high-resolution Fusobacterium detection in gut and tumor microbiota (20). Beyond znpA, alternative high-resolution markers—including rpoB and gyrB—have been identified to significantly aid detection technique development (24). Using TAS targeting these hypervariable loci (such as znpA) found that the main subspecies in dental plaque is Fnp, and the main subspecies in odontogenic abscess samples is Fna (71). However, TAS is still limited by primer design deviations and may miss unknown variant strains.

Digital PCR (ddPCR) technology has higher accuracy in detecting Fn subspecies. By using the principle of droplet separation, the detection sensitivity is increased to 0.1 copies/*µ*L, achieving 100% sensitivity and 50% specificity in Fn detection in fecal immunochemical test (FIT)-positive samples (72). It is particularly suitable for noninvasive screening of low-abundance strains in early CRC. This technology avoids the dependence on standard curves through absolute quantification, providing more reliable technical support for the typing detection of Fn. In the balance between technical practicality and economy, TaqMan probe-based qPCR has become the preferred choice in clinical practice due to its mature technical platform and multiplex detection capabilities. To date, various PCR-based methods targeting former subspecies have been established and robustly applied to clinical samples (73, 74). However, precaution must be taken in target selection, as recent observations indicate that conventional nusG-based primers are not specific to F. nucleatum sensu lato (73, 75) By designing subspecies-specific primers, qPCR can achieve absolute quantification of subspecies like Fna in a single reaction system, with a detection limit as low as 10 copies per reaction (76), For large-scale screening, qPCR costs are approximately one-fifth of ddPCR due to lower reagent expenses. However, the strict requirements for primer-probe design (requiring known target sequences) and the limitation of single-target detection necessitate complementation with NGS for complex microbiota analysis. More application scenarios are reflected in the use of NGS to screen out Fn subtype-specific molecular targets, then designing corresponding primer probes, and using qPCR technology to achieve cost-effective and high-precision Fn genotyping detection. When combined with constant-temperature amplification technology, highly sensitive on-site quantification can be achieved. Expand the noninvasive detection of CRC from clinical application to outpatient testing and home self-testing. At present, no Fn detection kit has obtained a Class III medical device registration certificate in NMPA or FDA. Products from biotechnology companies specializing in molecular diagnostics (e.g., Skygen, Biorigin, Creative Diagnostics), These products focus on qPCR-based detection of Fn DNA. However, these products are mainly designed for broad bacterial detection rather than subspecies-specific clinical diagnostics. Notably, major *in-vitro* diagnostic manufacturers have not yet developed Fn-specific clinical assays, reflecting the gap between research findings and standardized clinical applications. This limitation underscores the urgent need for regulatory-approved, subspecies-specific diagnostic platforms to enable widespread clinical adoption.

In conclusion, metagenomic advances such as MetaPhlAn, which substantially improve detection resolution at the species and strain levels, should be increasingly integrated into future research. Currently, NGS and TAS form the technical cornerstones of discovery research, while ddPCR and qPCR promote clinical transformation through their advantages in sensitivity and cost. The collaborative application of these four types of technologies not only improves the molecular typing system of Fn in CRC, but also reveals the deep connection between strain specificity and tumor heterogeneity, providing new molecular targets for individualized noninvasive diagnosis and treatment. As more Fn-typing reagents obtain regulatory approval, an increasing number of patients will benefit from more accurate auxiliary diagnosis of CRC via Fn detection.

The **Table 2** introduces the current representative Fn noninvasive detection technology methods and cases.

**Table 2:** The current representative Fn noninvasive detection technology methods and cases.

Method	Principle	Sample type	Advantages	Key studies
1. Metagenomic sequencing (mNGS)	Analyze Fn functional genes (such as Fap2, FadA)	Stool, Saliva	Enables concurrent detection of other microbiota at the same time advanced profilers (e.g., MetaPhlAn) now drastically improve species/strain level detection resolution.	Wirbel J. et al. (2019): Fn found to be associated with global microbial markers of CRC via metagenomic sequencing (77). Zepeda-Rivera, M. et al. (2024): Fna C2 subtype now dominates colonization in CRC and is related to tumor metabolism and invasiveness (19). Gu Y. et al. (2024): Analyzed metagenomic profiles of salivary and dorsal tongue microbiomes from an East-Asian cohort, identifying those bacterial taxa that were significant (70). Piccinno et al. (2025): Conducted a large-scale cross-cohort metagenomic study evaluating Fusobacterium signatures across diverse populations (78).
2.Targeted-amplicon sequencing	Perform deep sequencing of specific hypervariable genes (e.g., znpA, rpoB, gyrB). such as znpA	dental plaque	Subspecies composition can be quantitatively analyzed.	Krieger M. et al. (2024): Fnp is dominant in dental plaque, and Fna is dominant in odontogenic abscess samples (71). Bi et al. (2022): Developed FrpoB-seq for high-resolution Fusobacterium tracking in gut/tumor microbiota (20).
3.16S rRNA Gene Sequencing	Amplify the 16S rRNA V3-V4 region and identify Fn and its subspecies.	Stool, saliva, mouthwash, tongue coating	Enables concurrent detection of other microbiota.	Kelly CP, et al. (2024): the composition of the fecal bacterium of CRC patients was significantly different from that of non-CRC individuals (79). Flemer B. et al. (2018): Combining oral and fecal microbiota information for CRC and adenoma detection achieved high AUC values(0.94 and 0.98) (80). Camanes-Gonzalvo S, et al. (2024): Analyze saliva or oral samples (e.g., mouthwash, tongue coating, unstimulated saliva) to study the association with CRC (81).
4.Digital PCR (ddPCR)	Single-molecule absolute quantification with strong inhibitor resistance.	Stool	1,000-fold higher sensitivity than qPCR, ideal for low-abundance samples.	Datorre JG. Et al. (2024) Detection of Fn in FIT-positive samples forcolorectal cancer detection resulted in an AUC of 0.8203 (confidence interval (CI), 0.6464–0.9942), high sensitivity (100%), and specificity of 50% (72). Suehiro et al. (2017): In stool samples, the median copy number of Fn in the CRCgroup (158 patients), high-grade adenoma/carcinoma *in situ* group (19 patients), and low-grade adenoma group (11 patients) was significantly higher than that in the healthy control group (60 individuals) (82).
5.Quantitative polymerase chain reaction (qPCR)	Targeting Fn-specific genes for amplification and quantification (Note: conventional nusG-based primers lack specificity to F. nucleatum sensu lato).Targeting Fn-specific genes (such asnusG, nusA, and 16S rRNA) for amplification and quantification.	Stool, Saliva	High sensitivity (capable of detecting as low as 10 copies/*μ*L), rapid turnaround time, and cost-effectiveness.	Zhang X. et al. (2021): Developed a multiplex qPCR assay for detecting Fn DNA in saliva, revealing significantly elevated Fn levels in CRC patients (AUC = 0.841) (36). Komiya Y. et al. (2019): Identified identical Fn strains in both saliva and tumor tissues of CRC patients, supporting the oral-origin hypothesis (26). Zhong F. et al. (2025): Reported significantly higher relative expression levels of fadA and nusG in CRC fecal samples compared to normal controls (NC) (p < 0.0001) (83). Bi et al. (2021); Shen et al. (2025): Established and validated PCR-based assays targeting former subspecies in clinical samples (73, 74).

### 4.2 Clinical application as a noninvasive diagnostic marker for CRC

In recent years, research on Fn as a noninvasive CRC marker has gradually increased, and it has detection potential in a variety of sample types, such as stool and saliva. Quantitative detection of Fn DNA in stool can distinguish CRC patients from healthy controls (84). When this bacterial signal is combined with the routinely used FIT, the detection rate for both CRC and advanced adenoma is markedly enhanced (85). In one study, ddPCR technology was used to detect Fn DNA in stool samples, and the results showed that the proportion of high-level detection of Fn in FIT-positive samples was significantly higher than that in FIT-negative samples (47.2% vs 28.9%), and the presence of high levels of Fn in cancer patients was closely related to the presence of tumors (72). Combining Fn detection with FIT is currently the most promising application strategy and can significantly improve the early detection rate of CRC. In addition, other intestinal flora indicators can also be combined. For example, using the ratio of Fn to probiotics (such as Fn/Bb, Fn/Fp). The AUC for the diagnosis of stage I CRC can reach 0.804 and the sensitivity is 90.0%, showing the potential to detect early lesions (86).

As a completely noninvasive and easy-to-obtain sample, salivary Fn detection is an emerging research direction. Fn in saliva can indirectly reflect the status of CRC patients. Fn DNA levels in the saliva of CRC patients are significantly higher than those in healthy controls, patients with hyperplastic polyps, or those with adenomas. Importantly, even among individuals negative for conventional serum markers such as CEA or CA19-9, salivary Fn DNA retains a high diagnostic efficacy (AUC ≈ 0.83) (36), underscoring its additive clinical value. Additional cohorts have corroborated these findings, reporting AUCs of 0.841 in the training set and 0.860 in an independent validation set−performances that surpass those of traditional serum tumor markers (87). Beyond discrimination, salivary Fn DNA concentrations also correlate with lymph-node involvement and distant metastasis, offering a noninvasive window into metastatic risk (AUC = 0.763) (36). This shows that Fn can effectively screen for CRC under noninvasive conditions.

Therefore, Fn, as a potential diagnostic marker for CRC, can not only improve the early screening rate through stool testing, but also identify CRC patients earlier through saliva, providing new possibilities for noninvasive screening and providing an important reference for patients’ early diagnosis and treatment decisions. These research results not only provide a new perspective on the diagnostic methods of CRC, but also lay the foundation for future clinical applications.

### 4.3 Genotyping-guided prognostic risk stratification

Fn is closely related to the overall survival (OS) and disease-free survival (DFS) of CRC patients, accumulating evidence reveals that Fn abundance carries independent prognostic weight. High-level tissue or fecal Fn DNA is consistently linked to inferior overall survival (OS) and shorter disease-free survival (DFS) across stage-stratified cohorts, notably, in stage IV CRC, 5-year OS of Fn-positive patients reaches only half that of their Fn-negative counterparts (88), while recurrence rates climb from 11% to 21% (89). Even after restricting analysis to stages II−III, the survival gradient remains evident, implying that bacterial load refines risk stratification within traditionally defined risk groups (90).

Fn is also associated with tumor prognosis in different locations; Notably, patients with Fn-enriched right-sided colon cancer experience shorter PFS1 than other subgroups (9.7 months vs 11.2 months), and an Fn-positive right colon is further linked to shorter PFS2 (3.7 months vs. 6.7 months) (91). This phenomenon may be attributed to the correlation between Fn presence and reduced sensitivity to the cornerstone chemotherapy agent 5-fluorouracil (5-FU), thereby diminishing therapeutic response and ultimately compromising overall prognosis (92). Consequently, systematically measuring and targeting Fn and its related pathways promises to yield valuable insights into clinical management and may offer a new lever for improving the prognosis of colorectal cancer patients (84).

Overall, genotyping-informed risk models can earmark High-risk genetic subtype individuals for more aggressive adjuvant protocols and intensified surveillance, thereby channeling limited clinical resources toward those most likely to benefit. These data establish Fn genotyping and quantification as complementary tools for refining prognosis, guiding therapy intensity and enabling real-time surveillance in CRC care.

### 4.4 Targeted intervention and individualized treatment strategies based on classification

With the deepening of microbiome research, Fn has been identified as a key influencing factor in the occurrence, development and treatment response of colorectal cancer. The emergence of genotyping testing marks that Fn research has entered a new stage of precision medicine at the “strain level” or “subspecies level”, and its role in guiding targeted intervention and personalized treatment strategies for CRC has become increasingly prominent. Genotyping testing can predict a patient’s potential response to different treatment options in advance. Genotyping can predict treatment responses in two key ways. First, Fn abundance predicts chemotherapy resistance. Fn positivity is associated with oxaliplatin resistance (93). Different gene subtypes may play a more powerful role in these pro-resistance mechanisms. This strongly indicates that the patient may be insensitive to standard chemotherapy regimens based on 5-FU or oxaliplatin, thus prompting doctors to consider first-line combination drug strategies instead of waiting for chemotherapy to fail before adjusting. Another aspect of Fn can predict immunotherapy response. Fn is a key factor in shaping the tumor immune microenvironment. Fn directly binds to inhibitory receptors (such as TIGIT) on T cells and NK cells through its surface protein Fap2, thereby “turning off” the killing function of immune cells and creating an immunosuppressive microenvironment in preclinical study (94). This makes tumors less responsive to immune checkpoint inhibitors such as anti-PD-1/PD-L1 antibodies. However, emerging evidence indicates that Fn can also exert the opposite effect in specific contexts. Notably, recent studies demonstrate that Fn can paradoxically sensitize microsatellite stable (MSS) colorectal cancers, typically resistant to anti-PD-1 therapy. In patients with MSS CRC, high intratumoral levels of Fn have been associated with a more favorable response to anti-PD-1 treatment, suggesting that Fn may serve as a predictive biomarker of immunotherapy responsiveness (95). This apparent contradiction underscores the dual role of Fn as both a tumor promoting microbe and a potential enhancer of immune checkpoint inhibitor efficacy.

Based on the results of genotyping, clinical intervention strategies can be more targeted. The most straightforward strategy currently is the combined use of antibacterial and anticancer drugs. Some preclinical studies have pointed out that the use of Fn-sensitive antibiotics such as metronidazole is expected to reverse its mediated chemotherapy resistance and immunosuppression, thereby enhancing the effect of mainstream therapy (96). In addition, genotyping lays the foundation for the development of new and more precise therapies. For example, nanomedicine (Au@BSA-CuPpIX) that can specifically target Fn. The drug can accurately kill Fn under ultrasound activation without destroying other beneficial bacteria in another preclinical study (34). The unique surface antigens or metabolic pathways of specific subtypes revealed by genotyping are ideal targets for designing such highly specific targeted drugs.

The genotyping test of Fn has pushed the treatment of CRC from “single-dimensional” precision medicine based on the human genome to a new “dual-dimensional” era that combines the “human genome-tumor microbiome”. Genotyping provides a key tool for achieving truly personalized treatment by accurately identifying high-risk subtypes, predicting treatment response, and guiding targeted intervention. Despite these promising advances, significant challenges remain in standardizing detection methods, validating clinical utility across diverse populations, and fully elucidating the causal mechanisms underlying Fn-CRC associations.

## 5 Research challenges and future directions

### 5.1 Limitations of mechanism research

In the study of the interactions between Fn and CRC, there is a clear gap in research on existing mechanisms, which limits a comprehensive understanding of this complex relationship. Several gaps remain in our understanding: First, the transmission routes are unclear. According to the blood-borne diffusion hypothesis, intravenous injection of Fn was successful in colony formation in a mouse model of CRC (37), but the detection rate of Fn in human serum was only 3.3% (26). On the other hand, the theory of the oral-intestinal axis favors the entry of Fn into the intestine by ingestion, but its amount in the intestines of healthy people is extremely low (*<*0.1%) (19). Then there are still unexplained parts about the factors of colony formation. Although the Gal-GalNAc/Fap2 interaction has been confirmed as a mediator of adhesion (37), 30% of colorectal cancer tumors do not express Gal-GalNAc, and the involvement of unexplored adhesion factors (e.g., RadD, CbpF) is suspected (97). Finally, the molecular mechanisms of host-microbe interactions involve complex signaling networks and a lack of temporal and spatial dynamics in metabolic reprogramming. Inhibition of butyrate metabolism by Fn may promote colorectal cancer, but data to monitor metabolic flow *in vivo* are lacking (98). In addition, the heterogeneity of the Fn tribes poses major challenges. Fn strains from different sources exhibit significant genomic and pathogenicity variations that affect the reproducibility of research results. For example, some studies suggest that different subtypes of Fn may play a different role in CRC (99). Strain-dependent phenotypic differences were also observed. For example, the Fn strain 7−1 (isolated from IBD patients) causes colitis in mice, while the Fn strain ATCC 23726 (isolated from the oral cavity) does not show this effect (50). Moreover, the sensitivity to 5-FU shows an almost tenfold difference between the strains (96). Such heterogeneity can lead to significant inconsistencies when different laboratories try to replicate the same experiment. As Fn research progresses, more and more genomic data shed light on Fn’s different behaviors in different environments, making research into its role in this disease even more complex.

The debate over Fn’s causal role in CRC development remains unresolved−is it a “bystander” or a “driver”? Its specific role in early-stage lesions is also unclear. For instance, Fn is already enriched in adenomas (100), but it remains impossible to determine whether this is a cause or consequence. On the one hand, animal models lack sufficient humanization, and the gut microbiome of mice is markedly different from that of humans; On the other hand organoid models lack stromal components (fibroblasts, immune cells), which prevents the reproduction of Fn immunomodulation effects (101).

### 5.2 Clinical translation bottleneck: test standardization and specificity

Clinical translation of Fn−CRC associations is hindered by several interconnected obstacles. First, there is a lack of standardization in detection methods. For example, qPCR thresholds vary widely (tissue: 1.9−50 copies/ng DNA; stool: 10^3^ −10^6^ copies/g) (82, 100), and single-cell sequencing fails to distinguish intracellular Fn from extracellularly adhered bacteria (102). To address these inconsistencies, international initiatives like the Microbiome Quality Control (MBQC) project and Zhang et al. (2025) have already demonstrated that methodological standardization, particularly in sample processing and quantitative thresholds, was the most critical factor influencing diagnostic consistency across studies (103, 104)., however, there is currently a lack of standardized protocols for sample collection of Fn, and no external quality control programs or certified reference materials are available for this pathogen. Second, different marker genes yield incongruent trees, producing unstable and often conflicting assignments (105). Third, phenotypic schemes are uninformative when biochemical profiles overlap: the four recognized subspecies (Fnn, Fnp, Fnv, Fna) display near-identical enzyme activities and substrate utilization patterns, preventing reliable discrimination (17). Most critically, current subspecies categories fail to predict pathogenic potential. The pathogenic divergence between Fna C1 and C2, which was described in section 1.1, is not captured by existing taxonomic frameworks (19).

In addition, Fn lacks sufficient specificity to serve as a standalone CRC biomarker. The organism is enriched in both IBD and periodontitis, generating frequent false-positive results. Fecal Fn loads in IBD are 10- to 100-fold higher than in healthy controls and overlap the range observed in CRC (106). As a core constituent of dental plaque, Fn reaches 10^7^ CFU/mL in the saliva of severe periodontitis patients; identical strains have been recovered from oral biofilm and colorectal tumors in 42.9% of CRC cases (26). Seropositivity for Fn-IgG is 68% in CRC, but also 42% in IBD, yielding a specificity of only 58% (106). Whether the CRC-associated Fna clade is equally prevalent in IBD or periodontitis remains untested, further eroding diagnostic precision. This challenge is consistent with the broader CRC biomarker literature, in which promising noninvasive assays remain limited by selection bias, incomplete evaluation of precancerous lesions, insufficient cost-effectiveness data, and the need for large multicenter prospective validation before clinical adoption (107).

Comparative genomics indicates that virulence gene content, metabolite secretion profiles and immune-evasion strategies differ substantially between Fn isolates, implying that a single intervention will not benefit all carriers (108). Intratumor heterogeneity further compromises diagnostic accuracy. Multiregion sequencing reveals marked spatial variation in Fn abundance (p*<*0.001); a single biopsy misses the organism in 40% of truly infected lesions (109). Predilection for superficial tumor layers, with relative scarcity in deeper compartments, introduces sampling bias and inflates false-negative rates.

### 5.3 Multi-omics integrated analysis and new strategies for precision medicine

In recent years, with the rapid advancement of multi-omics technologies, researchers have begun integrating metagenomics, metabolomics, single-cell sequencing, and other approaches to investigate the role of Fn in the pathogenesis of CRC, particularly in promoting tumor advancement, therapy resistance, and inflammatory responses (49, 84, 100, 110).

The use of metagenomics allows researchers to characterize Fn-associated microbial communities and their relative abundance in CRC patients. Metagenomic analyses reveal complex interactions between Fn and other microorganisms, which may influence the formation and evolution of the TME (111). Concurrently, integrated metabolomics offers deeper insights into how Fn influences tumor metabolism and providing strong evidence for Fn as a potential biomarker. Fn can influence the metabolic pathways of host cells to stimulate the growth and movement of cancer cells. These include: (i) activation of glycolysis, promoting proliferation; (ii) enhancement of fatty acid metabolism, aiding tumor metastasis; and (iii) induction of autophagy, accelerating migration (54, 112, 113). Fn alters lipid metabolism, which changes the self-renewal ability of colorectal cancer stem cells, thus broadening our understanding of its role in carcinogenesis (114). This interference in the metabolic function not only alter cancer biology, but it can also induce drug resistance.

Single-cell sequencing technology could offer a new way to study TME cellular heterogeneity (115). Through single-cell RNA sequencing, it is possible to analyze gene expression in different populations of cells in tumors and find a population of cells induced by Fn (116). This technology helps us learn about how Fn modulates immune cell functions to aid tumor immune evasion mechanisms.

Precision medicine that tailor treatment to defined microbial subgroups, particularly Fn, is becoming increasingly important in colorectal cancer management. For Fn-positive tumors, adjunctive therapy with antibiotics can improve treatment outcomes by reducing bacterial load and reducing tumor aggressiveness (34, 117). If Fn is negative, other immune-based or targeted agents can be used (118). This dual classification not only improves prognosis but also influences clinical decision-making, thereby increasing accuracy and efficiency. Genome-wide analysis of Fn and its molecular interactions with host cells will identify additional potent targets. Fn promotes colorectal carcinogenesis through sustained proliferation, invasion and chronic inflammation (119). A further component of precision medicine is the longitudinal quantification of Fn in liquid biopsies (120). This minimally invasive approach permits real-time surveillance of the TME and provides an immediate read-out of therapeutic efficacy, enabling regimen adjustment to optimize clinical outcome.

Integrated multi-omic profiling combining metagenomics, metabolomics and single-cell sequencing furnishes a comprehensive view of the Fn−CRC axis. This unified analytical framework clarifies the multifactorial mechanisms through which Fn drives tumorigenesis, underscores the causal contribution of the microbiome to oncogenesis, and accelerates the development of patient-specific therapies anticipated to prolong survival and improve quality of life. Future investigations must further delineate the therapeutic relevance of Fn and establish robust pipelines for translating these data into routine clinical decision-making, thereby delivering genuine precision oncology.

## 6 Conclusion

The role of the microbiome, particularly the influence of Fn, has been increasingly recognized in the development and progression of CRC. With the proliferation of commercial research kits, the acceptance of Fn as an auxiliary diagnostic biomarker for CRC has been increasing. Although detection of Fn in tissue sections is accurate, its invasive nature limits its utility for large-scale screening. Therefore, this review focuses on noninvasive specimens. Through multi-omics integrated analysis, future research will provide new theoretical foundations and practical guidance for noninvasive early diagnosis and personalized treatment of colorectal cancer. However, research on Fn still faces many challenges.

Although the mechanisms of Fn-mediated carcinogenesis are partially understood, study results remain inconsistent. This diversity underscores the need to consider tumor heterogeneity and the influence of environmental factors must be comprehensively considered when interpreting the biological functions of Fn. Therefore, it is urgent to clarify the role (cause or consequence) of Fn in different CRC subtypes and different microenvironments through systematic mechanism studies in the future to provide a more accurate theoretical basis for targeted therapy. Current classification methods cannot effectively distinguish strain pathogenicity, and the origin of Fn in CRC tumors (oral vs. intestinal) remains unclear. This limits clinical translation, necessitating the establishment of more precise classification standards to ensure clear disease associations. Integrating multi-omics and epigenetic profiling broadens the characterization of Fn during colorectal cancer development, yet cost-effectiveness secures qPCR as the prevailing platform. Stable subspecies-specific markers remain scarce, and inconsistencies in taxonomic criteria amplify this limitation. Nevertheless, with deepening Fn research and the maturation of dedicated research-use reagents, pioneering submissions for regulatory approval of Fn detection kits are now under way and are expected to accelerate the clinical translation of noninvasive auxiliary diagnostics and precision management of CRC.

In summary, Fn plays a complex and important role in the occurrence and development of colorectal cancer. Although current research results are rich, further efforts are still needed in terms of detection standardization, accurate classification, mechanism analysis and clinical translation. Future research should pay attention to multidisciplinary collaboration and combine modern technological means to promote Fn-related research to a higher level, in order to open up broader prospects for early noninvasive diagnosis and precise treatment of CRC.

## Glossary

ANI: Average Nucleotide Identity
CRC: Colorectal cancer
ddPCR: Droplet Digital PCR
DFS: Disease-Free Survival
EMT: Epithelial-Mesenchymal Transition
FadA: Fusobacterium adhesin A
Fap2: Fusobacterium nucleatum Adhesin 2
FIT: Fecal Immunochemical Test
Fn: *Fusobacterium nucleatum*
Fna: *F. nucleatum* subsp. *Animalis*
Fnn: *F. nucleatum* subsp. *Nucleatum*
Fnp: *F. nucleatum* subsp. *Polymorphum*
Fnv: *F. nucleatum* subsp. *Vincentii*
FomA: Fusobacterium outer-membrane Protein A
Gal-GalNAc: Galactose-N-acetylgalactosamine
ICB: Immune Checkpoint Blockade
LPS: Lipopolysaccharide
MDSC: Myeloid-Derived Suppressor Cells
NK: Natural Killer
qPCR: Quantitative Polymerase Chain Reaction
RadD: Radical D
TAMs: Tumor-Associated Macrophages
TIGIT: T-cell immunoreceptor with Ig and ITIM domains
TME: Tumor Microenvironment
TILs: Tumor-Infiltrating Lymphocytes
Tregs: Regulatory T cells
